# Supplementary material for: The Influence of the Mixed DPC:SDS Micelle on the Structure and Oligomerization Process of the Human Cystatin C
Source: Membranes (Basel). 2020 Dec 24;11(1):17. doi: 10.3390/membranes11010017 (PMC7824358; doi:10.3390/membranes11010017)
Supplement: Supplementary file 1 [file membranes-11-00017-s001.pdf]

Supplementary Materials: The Influence of the Mixed DPC:SDS Micelle on the Structure and Oligomerization Process of the Human Cystatin C

Przemyslaw Jurczak [1,*], Emilia Sikorska [1], Paulina Czaplewska [2], Sylwia Rodziewicz-Motowidlo [1], Igor Zhukov [3,4,*] and Aneta Szymanska [1,*]

Supporting information

Table S1: The values of critical micelle concentrations in PBS buffer, at 22°C and 37°C, for the surfactants used in this study[1]

surfactant	CMC
dodecylphosphocholine (DPC)	1.36 mM (22°C)<br>1.39 mM (37°C)
sodium dodecyl sulfate (SDS)	1.1 mM (22°C)<br>1.27 mM (37°C)
DPC:SDS 5:1	0.65 mM and 1.18 mM (22°C)<br>0.74 mM (37°C)

[1]Sikorska, E. et al. Thermodynamics, size, and dynamics of zwitterionic dodecylphosphocholine and anionic sodium dodecyl sulfate mixed micelles *J. Therm. Anal. Calorim.* **2016**, *123*, 511–523.

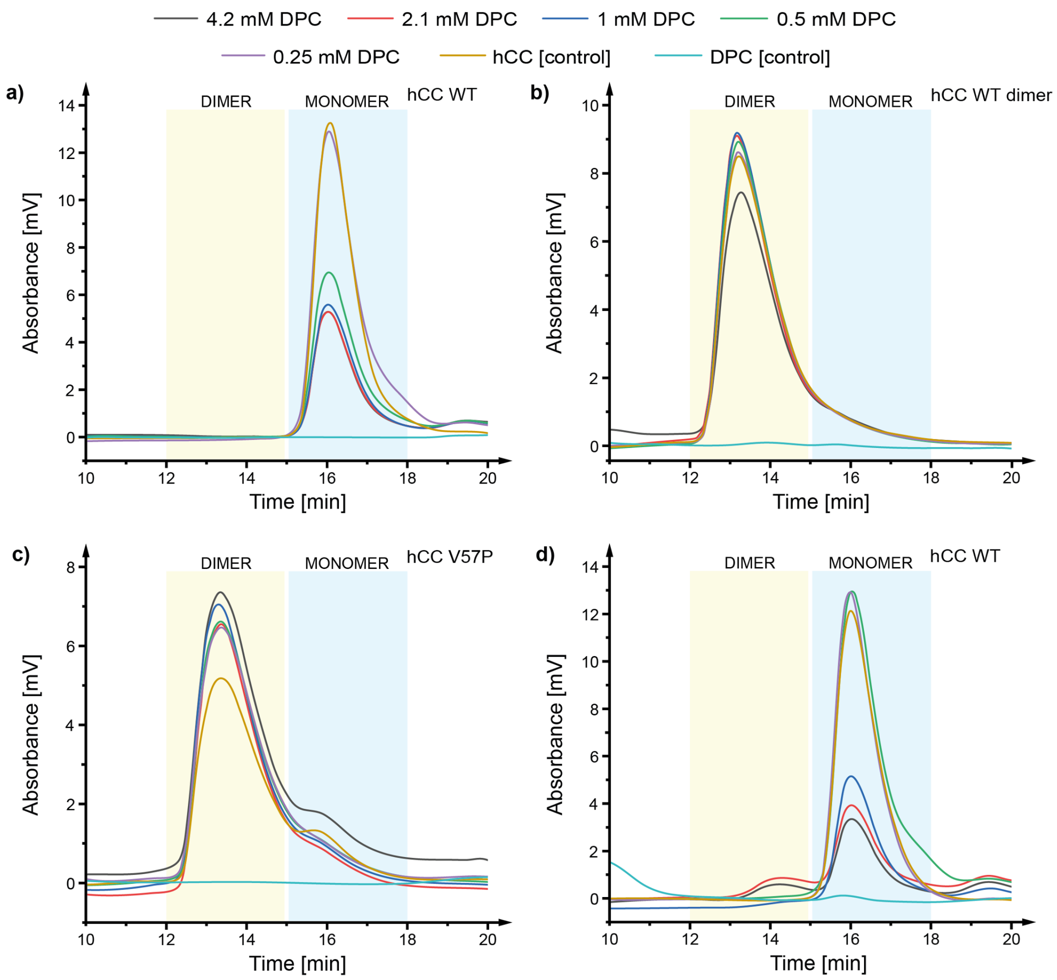

Figure S1. Chromatograms visualizing the separation of (a) *h*CC WT monomer, (b) WT dimer , and (c) V57P using the gel filtration chromatography after incubation for 24 h in the DPC solution at 22°C and (d) hCC WT monomer after incubation for 24 h in the DPC solution at 37°C (b); dimer retention time - ca. 13.5 min (yellow box), monomer retention time – ca. 16 min (blue box).

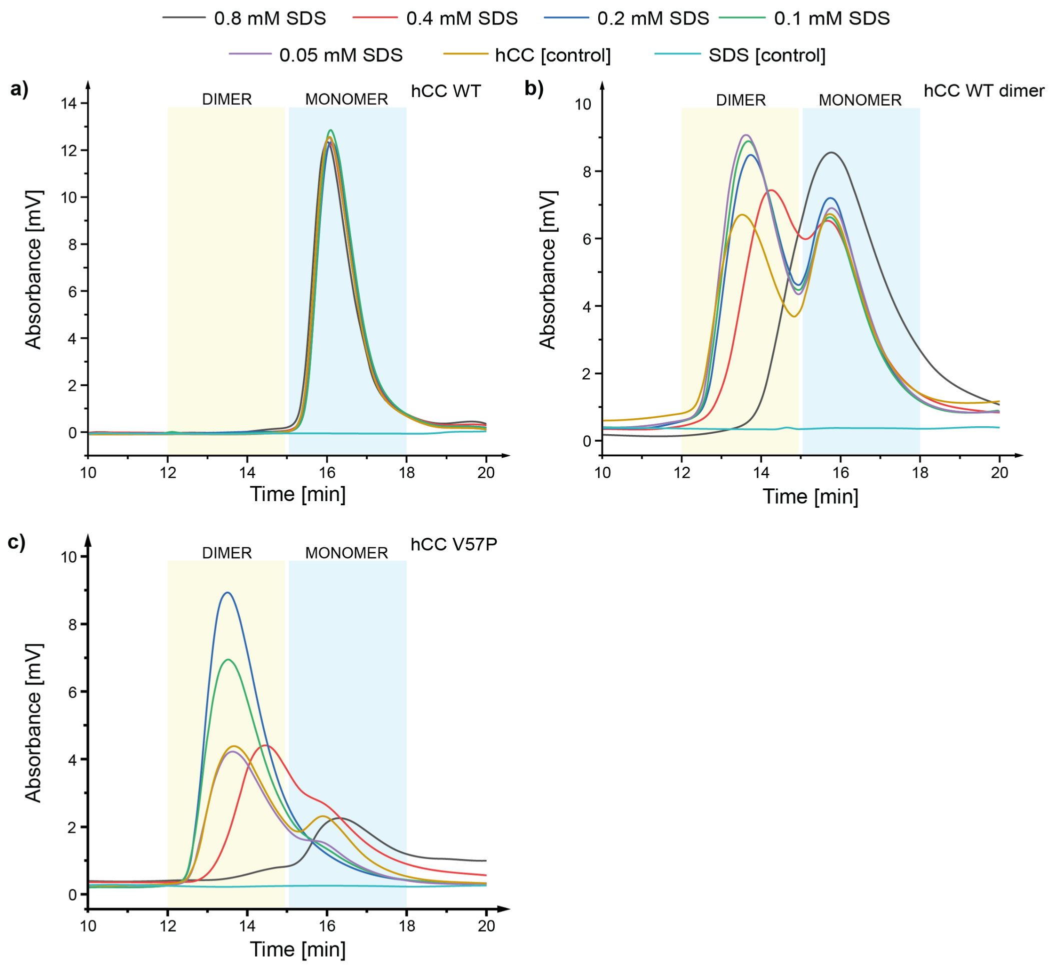

Figure S2. Chromatograms visualizing the separation of (a) *h*CC WT monomer, (b) *h*CC WT dimer and (c) *h*CC V57P using the gel filtration chromatography after incubation at 22°C for 24 h in the SDS solutions.

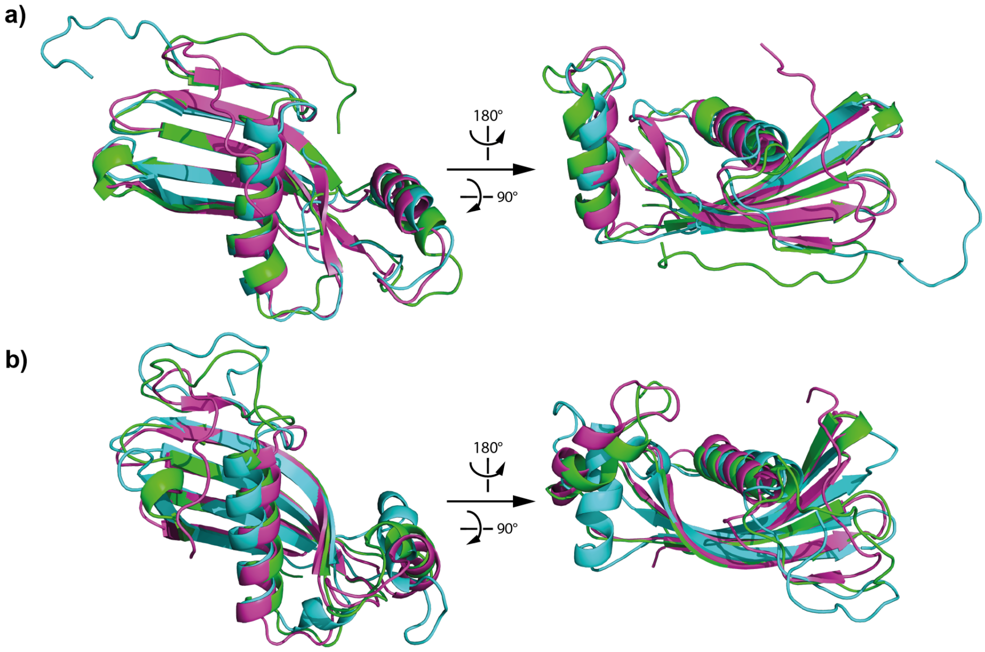

Figure S3. The structure of *h*CC V57G (a) before and (b) after 80 ns of MD simulation of *h*CC-DPC:SDS mixed micelle interaction; dynamics performed for three NMR structures differing mostly in the positioning of N-terminal part of the protein; NMR structure states: V57G-1 (green), V57G-2 (cyan), V57G-3 (magenta).

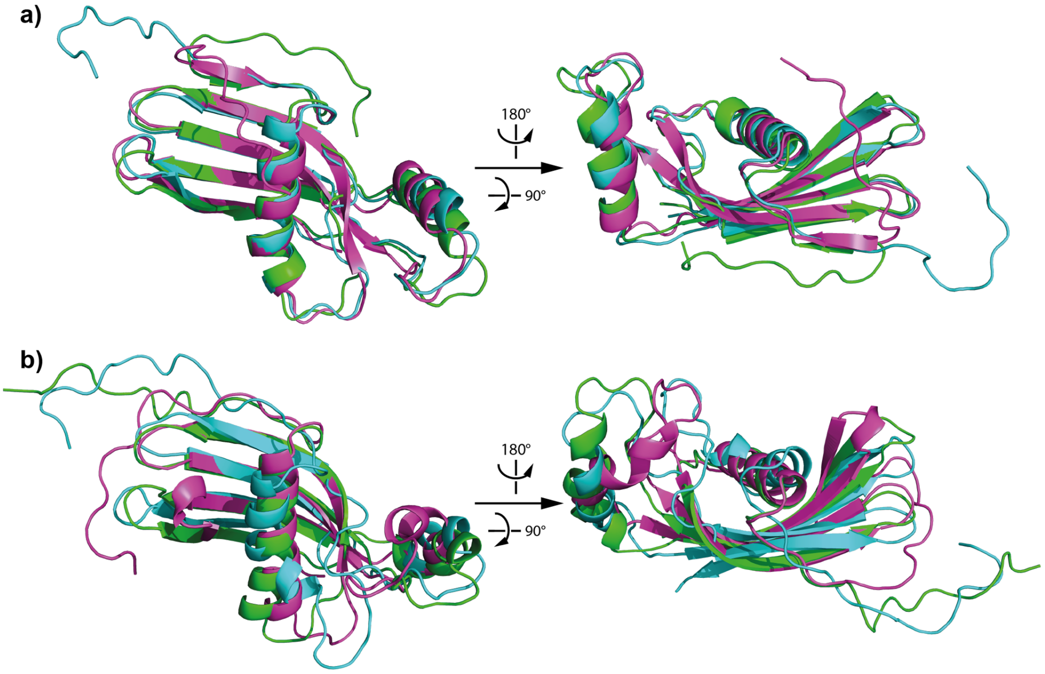

Figure S4. The structure of *h*CC WT (a) before and (b) after 80 ns of MD simulation of *h*CC-DPC:SDS mixed micelle interaction; dynamics performed for three NMR structures differing mostly in the positioning of N-terminal part of the protein; NMR structure states: *h*CC-1 (green), *h*CC-2 (cyan), *h*CC-3 (magenta).

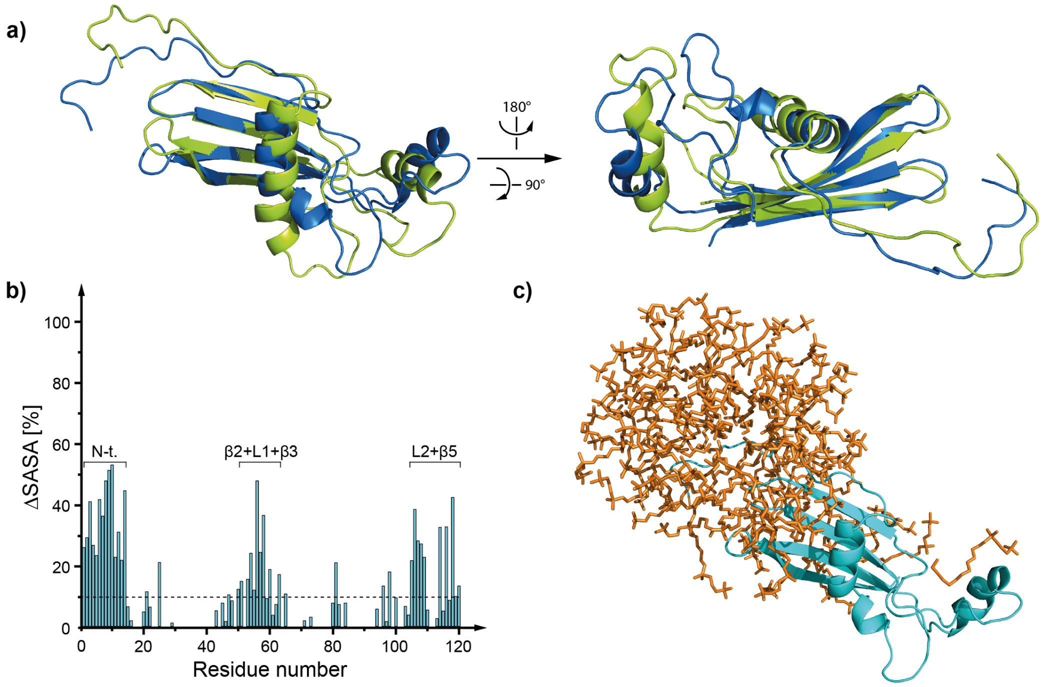

Figure S5. (a) The structure of *h*CC WT (*h*CC-2) monomer before (green) and after (blue) 80 ns of MD simulation of hCC-DPC:SDS mixed micelle interaction; (b) histogram visualizing the percentage of the decrease of solvent accessible surface occurring as a consequence of the interaction between *h*CC V57G monomer and DPC:SDS mixed micelle; (c) a model of *h*CC WT monomer protein interacting with the DPC:SDS mixed micelle, corresponding to the histogram (structure after 80 ns of MD); ΔSAS calculated as a difference between the SAS for the protein model without the micelle and protein model surrounded by the micelle.

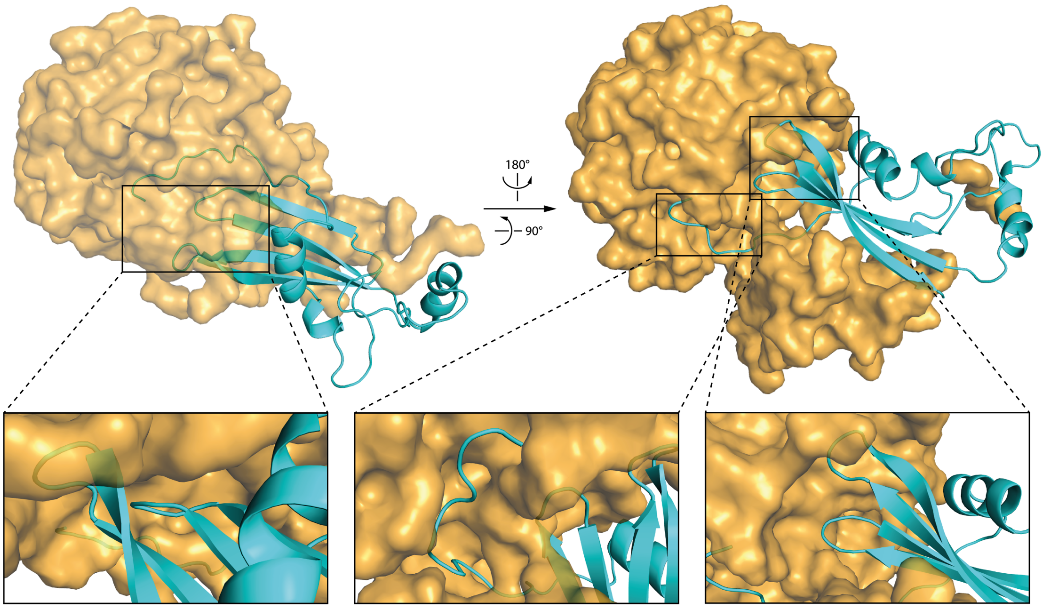

Figure S6. The structure of *h*CC WT (*h*CC-2) and DPC:SDS micelle complex after the MD simulations; insets present magnified fragments of the complex.

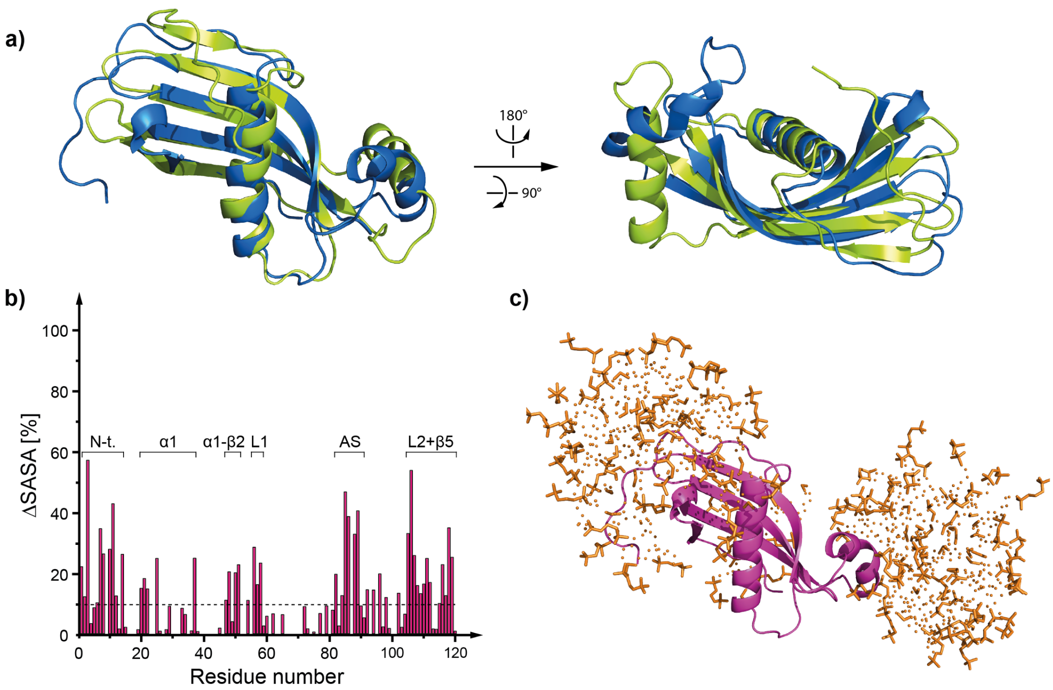

Figure S7. (a) The structure of *h*CC WT (hCC-3) monomer before (green) and after (blue) 80 ns of MD simulation of *h*CC-DPC:SDS mixed micelle interaction; (b) histogram visualizing the percentage of the decrease of solvent accessible surface occurring as a consequence of the interaction between *h*CC V57G monomer and DPC:SDS mixed micelle; (c) a model of *h*CC V57G monomer protein interacting with the DPC:SDS mixed micelle, corresponding to the histogram (structure after 80 ns of MD); ΔSAS calculated as a difference between the SAS for the protein model without the micelle and protein model surrounded by the micelle.

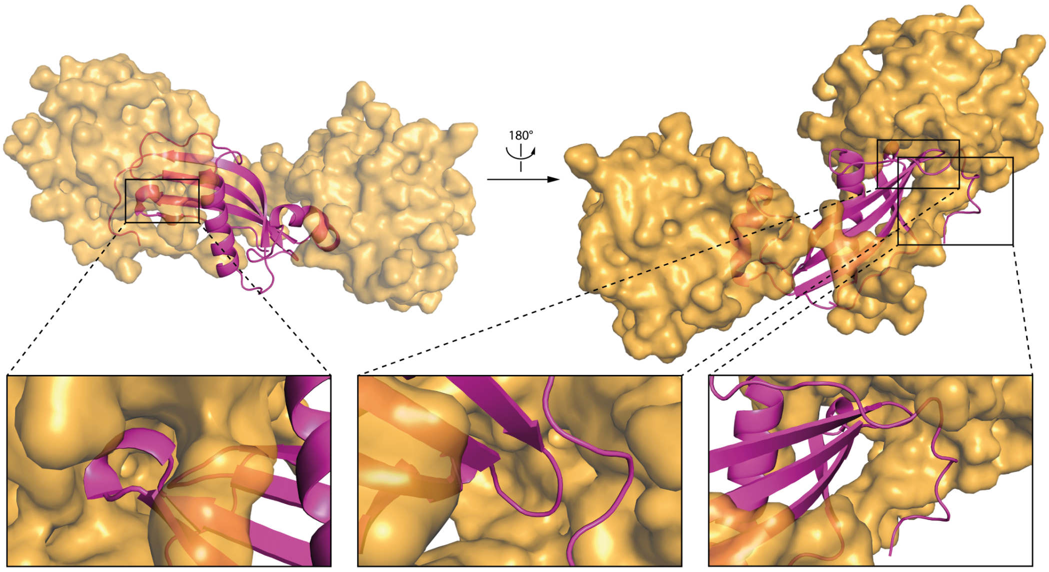

Figure S8. The structure of *h*CC WT (*h*CC-3) and DPC:SDS micelle complex after the MD simulations; insets present magnified fragments of the complex.

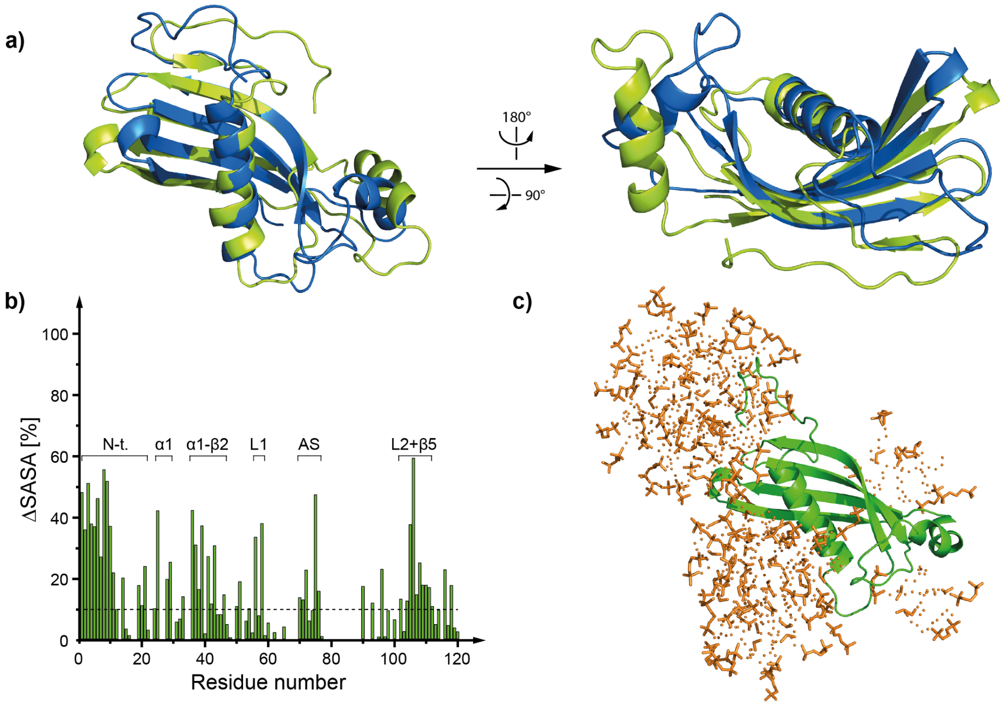

Figure S9. (a) The structure of *h*CC V57G (V57G-1) monomer before (green) and after (blue) 80 ns of MD simulation of *h*CC-DPC:SDS mixed micelle interaction; (b) histogram visualizing the percentage of the decrease of solvent accessible surface occurring as a consequence of the interaction between *h*CC V57G monomer and DPC:SDS mixed micelle; (c) a model of *h*CC V57G monomer protein interacting with the DPC:SDS mixed micelle, corresponding to the histogram (structure after 80 ns of MD); ΔSAS calculated as a difference between the SAS for the protein model without the micelle and protein model surrounded by the micelle.

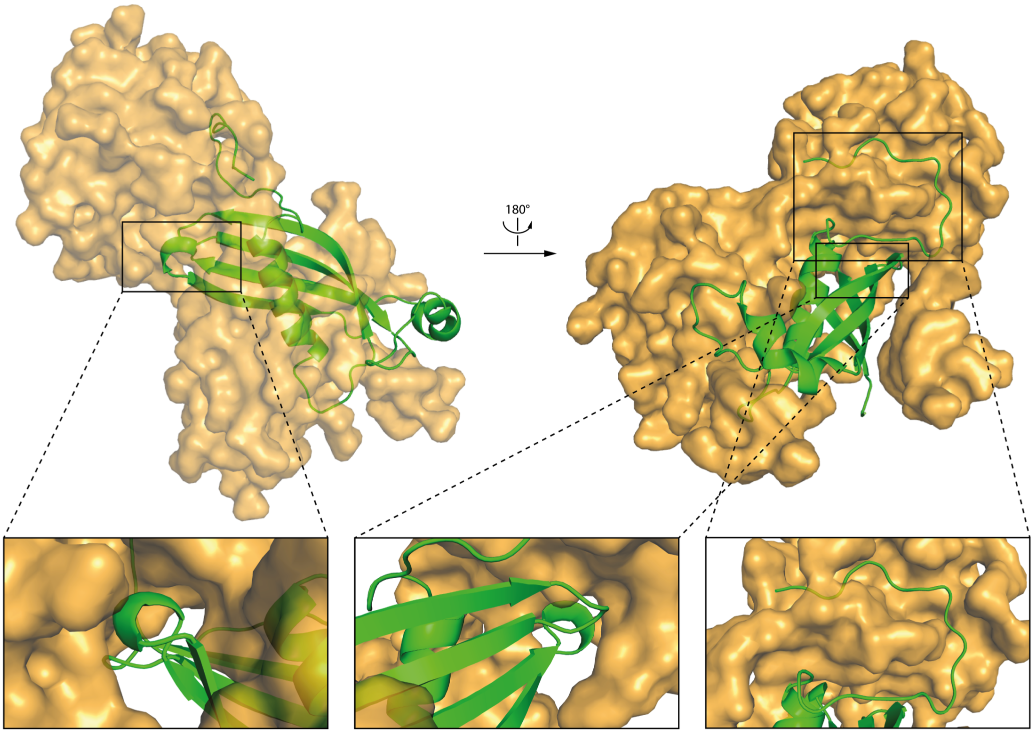

Figure S10. The structure of *h*CC V57G (V57G-1) and DPC:SDS micelle complex after the MD simulations; insets present magnified fragments of the complex.

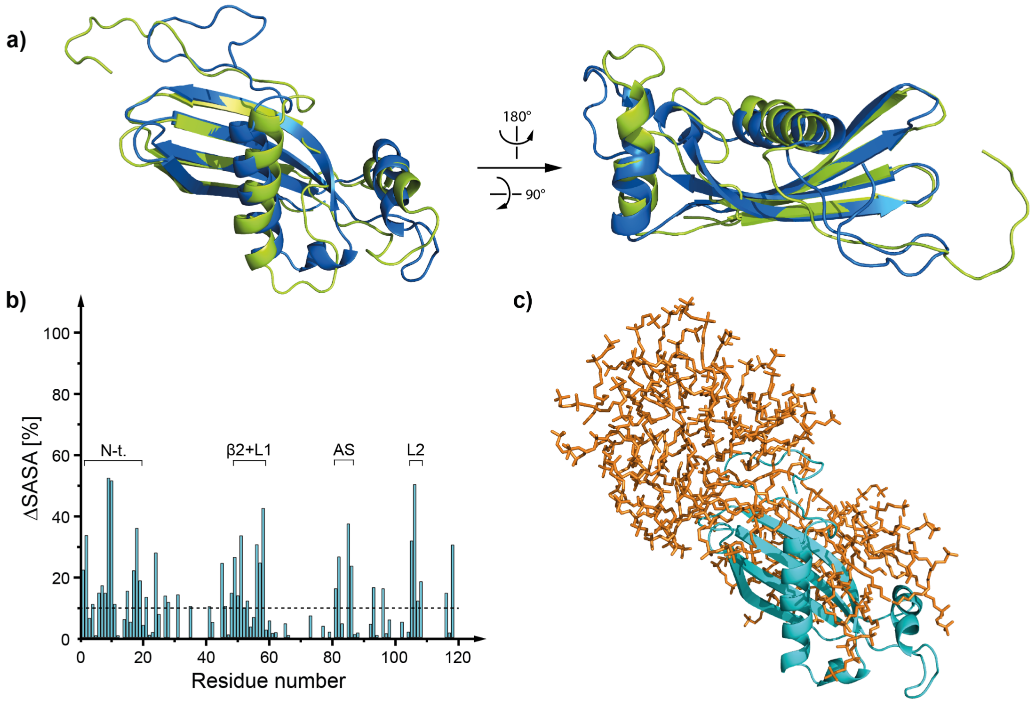

Figure S11. (a) The structure of *h*CC V57G (V57G-2) monomer before (green) and after (blue) 80 ns of MD simulation of *h*CC-DPC:SDS mixed micelle interaction; (b) histogram visualizing the percentage of the decrease of solvent accessible surface occurring as a consequence of the interaction between *h*CC V57G monomer and DPC:SDS mixed micelle; (c) a model of *h*CC V57G monomer protein interacting with the DPC:SDS mixed micelle, corresponding to the histogram (structure after 80 ns of MD); ΔSAS calculated as a difference between the SAS for the protein model without the micelle and protein model surrounded by the micelle.

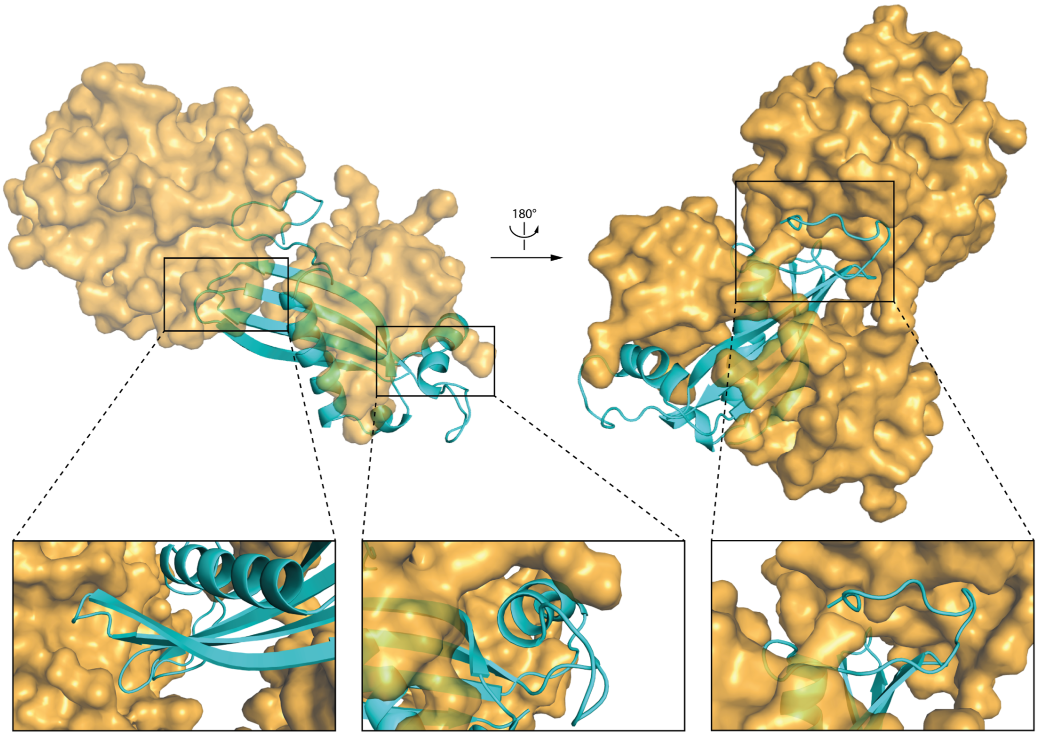

Figure S12. The structure of *h*CC V57G (V57G-2) and DPC:SDS micelle complex after the MD simulations; insets present magnified fragments of the complex.

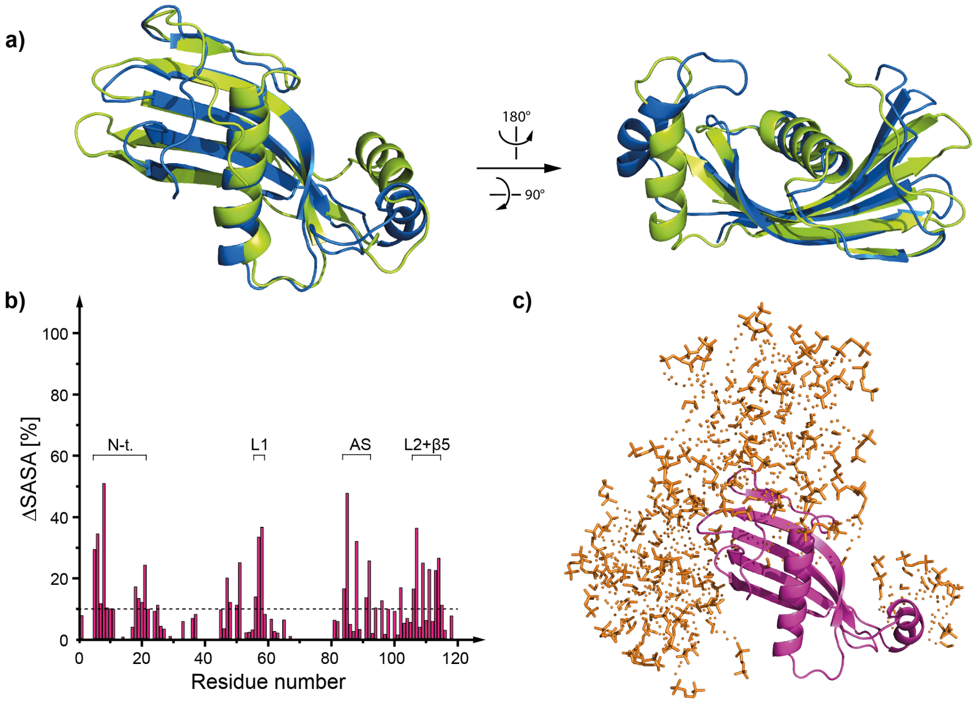

Figure S13. (a) The structure of *h*CC V57G (V57G-3) monomer before (green) and after (blue) 80 ns of MD simulation of *h*CC-DPC:SDS mixed micelle interaction; (b) histogram visualizing the percentage of the decrease of solvent accessible surface occurring as a consequence of the interaction between *h*CC V57G monomer and DPC:SDS mixed micelle; (c) a model of *h*CC V57G monomer protein interacting with the DPC:SDS mixed micelle, corresponding to the histogram (structure after 80 ns of MD); ΔSAS calculated as a difference between the SAS for the protein model without the micelle and protein model surrounded by the micelle.

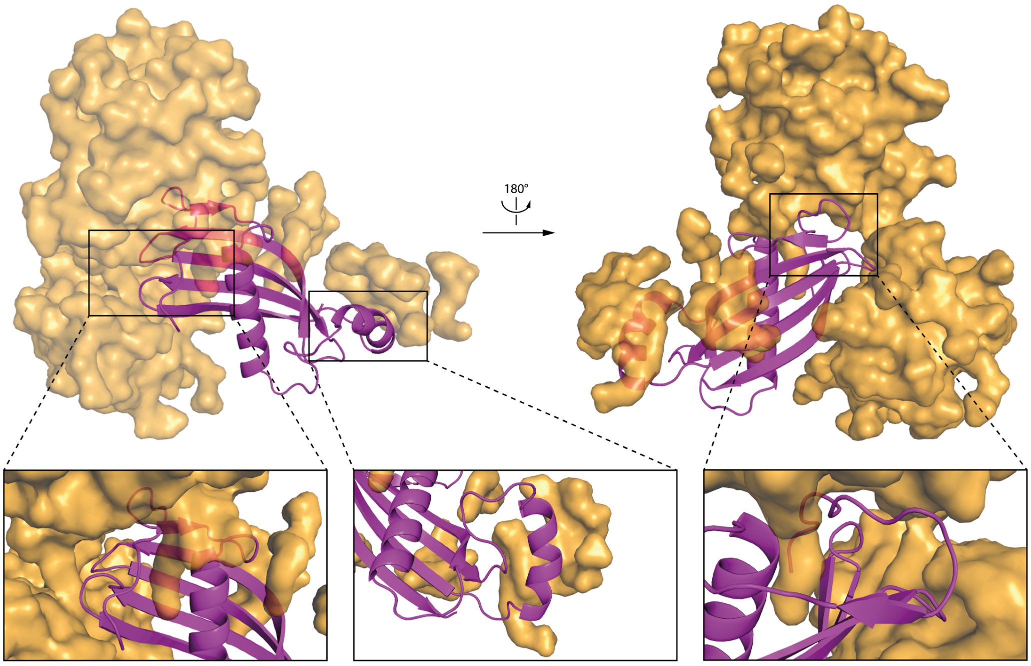

Figure S14. The structure of *h*CC V57G (V57G-3) and DPC:SDS micelle complex after the MD simulations; insets present magnified fragments of the complex.

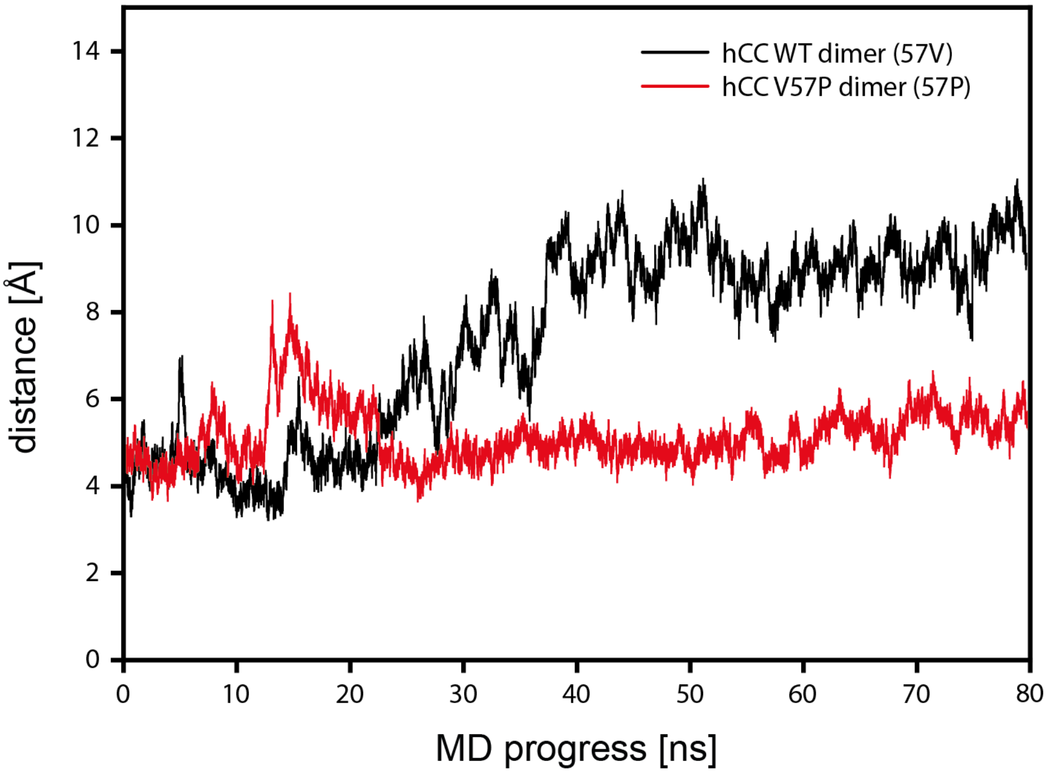

Figure S15. Changes in the distance between mass centers of domains in the *h*CC dimers during MD simulations; the comparison for *h*CC WT and *h*CC V57P dimers.

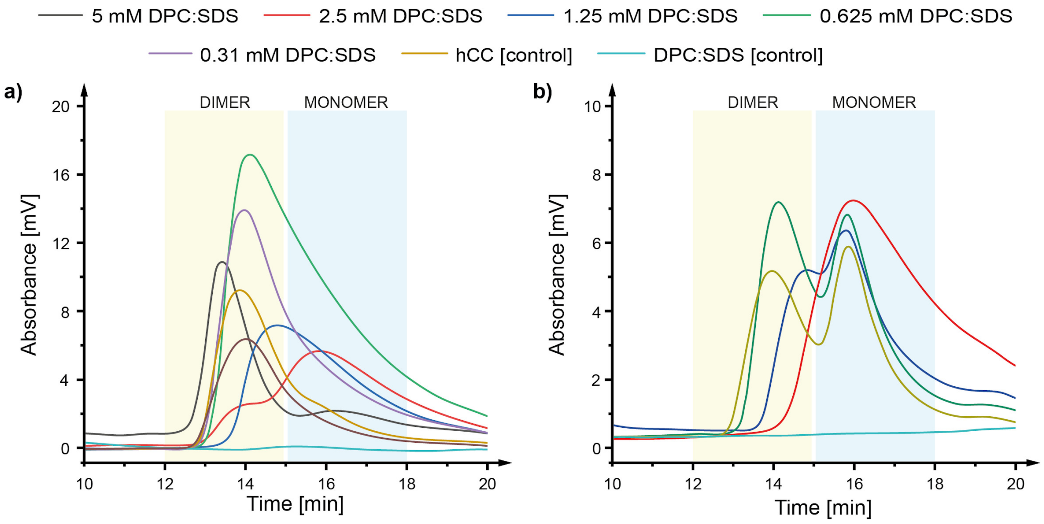

Figure S16. Chromatograms visualizing the oligomerization state of **(a)** *h*CC WT dimer and **(b)** a mixture of *h*CC WT monomer:dimer (1:1 molar ratio), using the gel filtration chromatography after incubation at 22°C for 24 h in the DPC:SDS (5:1) mixed micelle solution; *h*CC V57P was used as a dimer marker in **(a)**; dimer retention time – ca. 13.5 min (yellow box), monomer retention time – ca. 16 min (blue box).

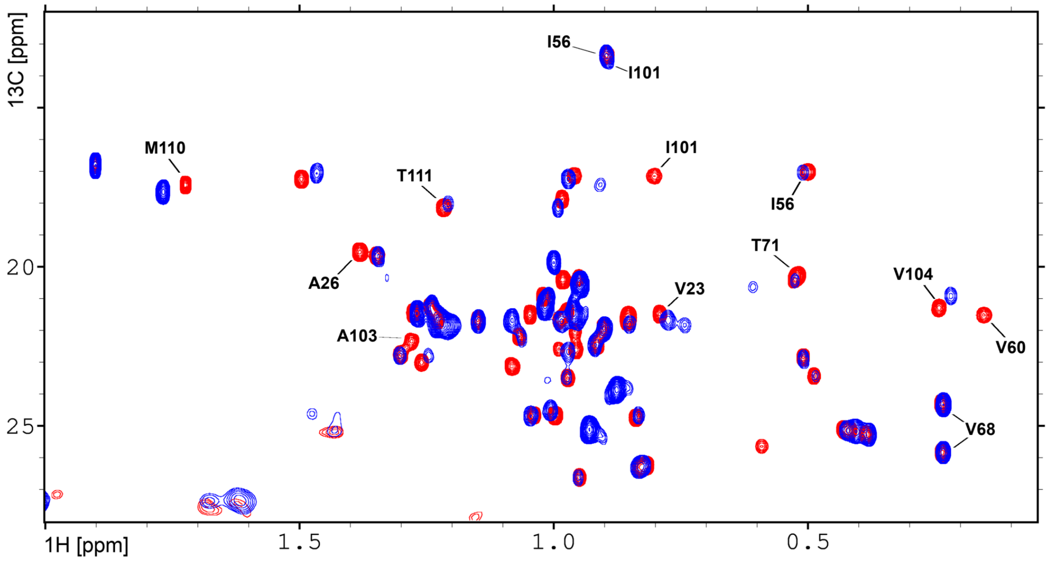

Figure S17. Overlay of 2D ^{1}H-^{13}C HSQC spectra (fragment) presenting signals from methyl groups recorded for *h*CC V57G protein only (red) and in the presence of the DPC-d_{38}:SDS-d_{25} micelle (blue). The changes in the position of cross peaks detected for a couple of residues. The increased linewidth in ^{1}H dimension observed for some signals resulting from the interaction with the DPC-d_{38}:SDS-d_{25} micelle corresponds to the increased molecular mass.
